# Remote Sensing Phenology of Antarctic Green and Red Snow Algae Using WorldView Satellites

**DOI:** 10.3389/fpls.2021.671981

**Published:** 2021-06-16

**Authors:** Andrew Gray, Monika Krolikowski, Peter Fretwell, Peter Convey, Lloyd S. Peck, Monika Mendelova, Alison G. Smith, Matthew P. Davey

**Affiliations:** ^1^Department of Plant Sciences, University of Cambridge, Cambridge, United Kingdom; ^2^Field Spectroscopy Facility (Natural Environment Research Council), University of Edinburgh, Edinburgh, United Kingdom; ^3^British Antarctic Survey, Natural Environment Research Council, Cambridge, United Kingdom; ^4^The Scottish Association for Marine Science, Oban, United Kingdom

**Keywords:** snow algae, Antarctica, remote sensing, snow, satellites, WorldView, ecology

## Abstract

Snow algae are an important group of terrestrial photosynthetic organisms in Antarctica, where they mostly grow in low lying coastal snow fields. Reliable observations of Antarctic snow algae are difficult owing to the transient nature of their blooms and the logistics involved to travel and work there. Previous studies have used Sentinel 2 satellite imagery to detect and monitor snow algal blooms remotely, but were limited by the coarse spatial resolution and difficulties detecting red blooms. Here, for the first time, we use high-resolution WorldView multispectral satellite imagery to study Antarctic snow algal blooms in detail, tracking the growth of red and green blooms throughout the summer. Our remote sensing approach was developed alongside two Antarctic field seasons, where field spectroscopy was used to build a detection model capable of estimating cell density. Global Positioning System (GPS) tagging of blooms and *in situ* life cycle analysis was used to validate and verify our model output. WorldView imagery was then used successfully to identify red and green snow algae on Anchorage Island (Ryder Bay, 67°S), estimating peak coverage to be 9.48 × 10^4^ and 6.26 × 10^4^ m^2^, respectively. Combined, this was greater than terrestrial vegetation area coverage for the island, measured using a normalized difference vegetation index. Green snow algae had greater cell density and average layer thickness than red blooms (6.0 × 10^4^ vs. 4.3 × 10^4^ cells ml^−1^) and so for Anchorage Island we estimated that green algae dry biomass was over three times that of red algae (567 vs. 180 kg, respectively). Because the high spatial resolution of the WorldView imagery and its ability to detect red blooms, calculated snow algal area was 17.5 times greater than estimated with Sentinel 2 imagery. This highlights a scaling problem of using coarse resolution imagery and suggests snow algal contribution to net primary productivity on Antarctica may be far greater than previously recognized.

## 1. Introduction

Blooms of microalgae were noted in Antarctic snow fields by several expeditions in the early 1900s, in a period when scientific discovery became a major driving force for exploration of the continent (Hirano, 1965). Since these early records, blooms of red, green, and orange snow algae in Antarctica have been revealed as diverse ecosystems that play an active role in biogeochemical cycling of nutrients and carbon (Ling and Seppelt, 1993; Dierssen et al., 2002; Hodson et al., 2008; Remias et al., 2013; Boetius et al., 2015; Davey et al., 2019; Procházková et al., 2019). Antarctica has relatively little exposed land to support terrestrial vegetation, with 98.7% of its surface area permanently covered in snow or ice (Fretwell et al., 2011). Thus, growth within the interstitial water of melting snow may be a successful, or even dominant, strategy for photosynthetic life there. Indeed, previous work has identified green snow algal blooms covering 1.9 km^2^ of the Antarctic Peninsula within a melt season (Gray et al., 2020), which compares with standing area estimates for other terrestrial vegetation of between 2.44 and 44.6 km^2^ (depending on the level of certainty) (Fretwell et al., 2011). Monitoring snow algae is important in the face of warming in Antarctica, as snow loss from low-lying islands competes with increases in snow melt at higher elevations to influence the area and distribution of habitable snow (Gray et al., 2020). Moreover, snow algae are impurities within the snowpack, absorbing more solar radiation than clean snow, reducing its albedo and enhancing melt (Lutz et al., 2016; Ganey et al., 2017; Khan et al., 2021). The study of snow algae in Antarctica, in particular optimizing approaches to accurately monitor their ecology and influence on snow albedo, is timely and crucial to improve understanding of the effect of climatic changes on their distribution and abundance.

Satellite remote sensing greatly increases our ability to map and monitor the extent of Antarctica's terrestrial biosphere, because other approaches, such as airborne or drone-based remote sensing missions cover relatively small areas and are geographically tethered to research station infrastructure. Satellite imagery has provided valuable insight into the distribution and biomass of terrestrial vegetation in Antarctica (Fretwell et al., 2011; Casanovas et al., 2015; Jawak et al., 2019), but these studies do not include snow algae as their spectral reflectance profile, measured using field spectroscopy (Painter et al., 2001; Cook et al., 2017; Huovinen et al., 2018; Di Mauro et al., 2020; Gray et al., 2020), is not identified using classical vegetation indices. There are, however, several studies that focus specifically on remote sensing snow algae, from early work using airborne hyperspectral imaging (Painter et al., 2001), projects utilizing satellite observations (Takeuchi et al., 2006; Huovinen et al., 2018; Tedstone et al., 2019; Di Mauro et al., 2020; Gray et al., 2020), and drone-based observations (Tedstone et al., 2019). Each technology has advantages and disadvantages, with airborne sensors giving high spatial and spectral resolution across moderate areas, satellites offering relatively coarse spatial and spectral resolution across large areas, and drones offering custom spectral resolution at very high resolutions, but over small areas.

Our previous work mapping snow algae on the Antarctic Peninsula used the freely available European Space Agency's (ESA) Sentinel 2 satellite imagery and gave the first insight into the distribution of blooms in Antarctica over a large scale (Gray et al., 2020). However, the spatial resolution of satellite imagery was too coarse to capture blooms at the edges of snow patches, and importantly, it was not able to determine the presence of blooms of red snow algae, as their secondary carotenoids reduce the effectiveness of the spectral index used. Moreover, this analysis focused only on a snapshot of growth from 1 year's worth of imagery at each bloom location. The problem of red bloom detection in Sentinel 2 imagery has been somewhat addressed by the work of Khan et al. (2020), yet large uncertainties remain when deriving area and biomass estimates from 10 m spatial resolution pixels. Key to using remotely sensed observations to study Antarctic terrestrial ecology lies in this balance between spatial resolution and area coverage. Using higher resolution commercial satellite imagery may help to address some of the uncertainties of coarser resolution Sentinel 2 or Landsat imagery, yet it can also be used to study blooms over larger areas.

Here, we apply the Painter et al. (2001)-adapted methodology from Gray et al. (2020) to high spatial resolution WorldView 2 and 3 satellite imagery to study the ecology of snow algae in Antarctica. WorldView satellites (Maxar Technologies) offer some of the highest spatial and spectral resolution satellite imagery that are commercially available and have shown great utility for studying Antarctica's ecology (LaRue et al., 2011; Fretwell et al., 2012; Cubaynes et al., 2019; Jawak et al., 2019). We aimed to assess the use of high-resolution optical satellite remote sensing as a tool to study snow algae, and in so doing improve upon estimates of area, biomass, and uncertainty from our earlier study (Gray et al., 2020). The band configuration of WorldView satellites enabled us to adapt our earlier methodology to include red snow algae in our analysis, and higher spatial resolution (1.24 m^2^) pixels meant we could improve our understanding of spatial scale when deriving remotely sensed ecological measures for snow algae. Finally, we use WorldView imagery to study the phenology of snow algae over a growth season in the Ryder Bay area of Adelaide Island, Antarctica.

## 2. Materials and Methods

**Figure 1:**
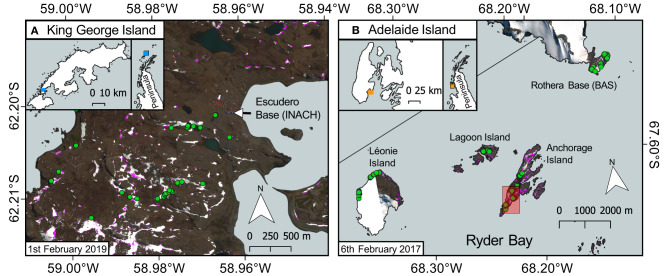
Ground validation sites. WorldView 3 red–green–blue (RGB) images of our ground validation sites, the extent and location of which are denoted by the blue- or orange-shaded areas in their respective inset maps. Green circles show locations where snow algae were visibly growing on the snow surface, were geotagged, measured with the field spectrometer, and sampled for microscopy analysis. Pink pixel shading denotes areas of snow identified as containing snow algae within those WorldView 3 images, using the approach outlined below. **(A)** Sampling conducted in the Fildes Peninsula region of King George Island whilst stationed at Professor Julio Escudero Station and Refugio Collins in 2019. **(B)** Sampling conducted in the Ryder Bay area of Adelaide Island, showing Rothera Research Station and the outlying islands from which snow algae were sampled in 2018. Red shaded box on Anchorage Island denotes the area shown in Figures 9, 12. Map data from the SCAR Antarctic Digital Database.

Fieldwork was conducted in the Fildes Peninsula area of King George Island (62°S) in January and February 2019 (Figure 1A) and the Ryder Bay (Rothera Research Station) area (67°S) in January and February 2018 (Figure 1B). Parallel work packages were undertaken to collect information on snow algal life cycle, collect field spectrometer measurements to build our remote sensing model, and to record the locations of blooms for later validation of satellite imagery. WorldView 3 imagery was tasked for collection during both field campaigns. Adverse cloud conditions on collection days meant that only one image was collected coincident to fieldwork, from February 1, 2019 on King George Island, and it suffers from over-saturation within the blue and green bands. Though we were able to use this image to partially verify our approach, the major remote sensing discussion herein is focused around a mixture of WorldView 2 and 3 imagery of Ryder Bay, captured in 2017, with images from February 6, February 28, November 23, and December 28.

### 2.1. Field Studies

#### 2.1.1. Snow Algal Bloom Sampling

Previous work in the Ryder Bay area has shown green coloration of snow, mostly resulting from *Chloromonas, Chlamydomonas*, and *Chlorella* sp., in the slush layer between multiyear and seasonal snowpacks (Davey et al., 2019). Red coloration, largely a result of *Chloromonas* sp., was also visible on the surface of seasonal snow (Davey et al., 2019). This stratification has been observed elsewhere in Antarctica, with green or orange coloration at depth (Bidigare et al., 1993; Mataloni and Tesolín, 1997; Remias et al., 2013; Hodson et al., 2017) as well as red/orange/pink coloration on the snow surface (Fujii et al., 2010; Segawa et al., 2018; Procházková et al., 2019; Soto et al., 2020).

**Figure 2:**
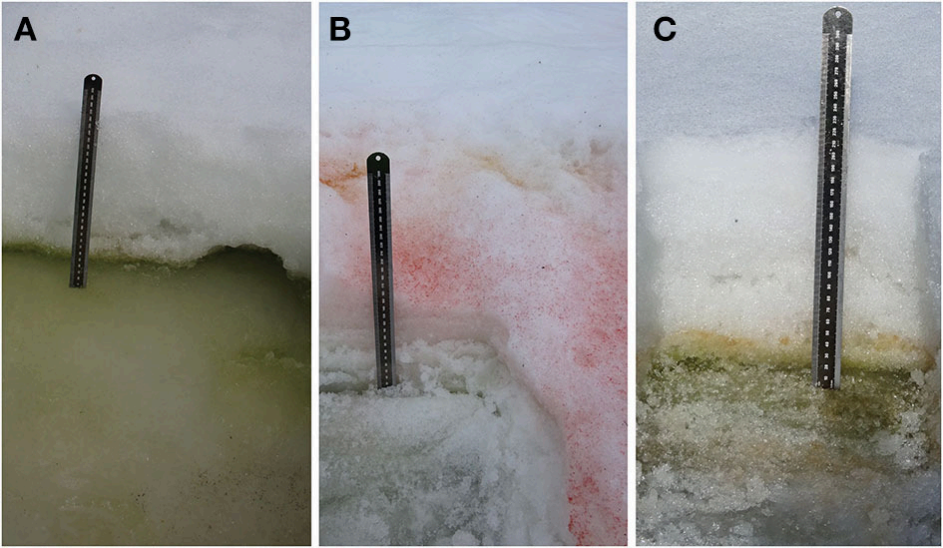
Snow algae pits. Photographs showing **(A)** a green bloom underneath seasonal snow layer. Lagoon Island, January 12, 2018; **(B)** a red bloom on the surface of seasonal snow. Léonie Island, January 30, 2018; **(C)** red algae emerging on top of band of green snow algae. Anchorage Island, February 9, 2018.

Six blooms, in which snow was visibly colored red or green, were identified close to Rothera Research Station (Rothera Point) and on the nearby islands of Anchorage, Lagoon, and Léonie. GPS locations and photographs of the blooms were taken. Within the bloom, three sampling patches (sample pits) were identified, one central and one to the left and right of center, and carefully dug out with a snow shovel (pits are shown in Figure 2). One patch/pit with no visible snow algae was also sampled to provide a negative control at each location, close to the visible bloom area. At each patch, we sampled the surface white (or sometimes green or red algal top layer) snow, the algal layer (usually green), and the underlying snow or ice layer. The algal layers were sampled using 4 × 50 mL (28.98 cm^2^) sterile plastic sample tubes and analyzed according to the methods described in Davey et al. (2019) and Gray et al. (2020). Briefly, the depth of layer was recorded as well as photosynthetically active radiation (PAR) (Skye PAR Quantum Sensor, Skye Instruments Ltd., Powys, UK) and temperature (Omega HH306A thermometer; OMEGA Engineering Inc) at the snow surface and algal layers at the start of each sampling period. The samples were then transferred to the Bonner Laboratory (Rothera Research Station, Ryder Bay, Antarctica) for processing. Within the research station, samples were melted in 4°C lit incubators (Sanyo). Algal cell density was measured in 202 samples, by agitating and adding 6 μl of snowmelt into Hycor Kova® hemocytometer wells and counting the number of algal cells using bright field microscopy or adding a known volume (10–20 μL) to a slide, drying, and counting all the algal cells. Algal community dry cell mass was obtained by gravity filtration of 50 mL of melted snow through a pre-weighed dry filter (Whatman GF/C, 47 mm). Filters were dried at 80°C for at least 48 h prior to re-weighing.

#### 2.1.2. Remote Sensing Model Development and Validation

*In situ* hemispherical directional reflectance factors (HDRF) of green (*n* = 91) and red (*n* = 57) snow algae, control white snow patches (*n* = 38), and snow containing mineral dust but no algae (*n* = 36) were recorded at the locations noted by green circles in Figure 1. Spectra were collected under clear sky conditions with a Spectra Vista Corporation (SVC) 1024i field spectrometer using the methodology described in Gray et al. (2020). Fixed viewing geometry ensured that HDRF was recorded consistently over a 908 cm^2^ field of view (FOV) (roughly 6% the area of a WorldView 3 pixel). The snow in the FOV was sub-sampled into a sterile 50 mL sample tube, with care taken not to compress the snow into the tube. The samples were then transferred to the Bonner laboratory or the Professor Julio Escudero Base laboratory [King George Island (KGI), Antarctica], where cell density was determined through analysis of color brightfield microscope images using the methodology described in Gray et al. (2020). Field samples were collected at Ryder Bay under the UK BAS Operating Permit and the Antarctic Act (1994; 2013) and at KGI under permit from INACH (Chile) Certificate number 209/2019.

**Figure 3:**
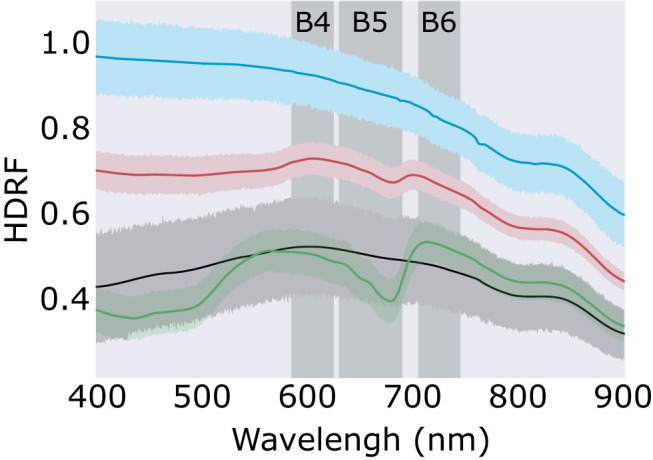
Snow algae reflectance factors. Average hemispherical directional reflectance factors (HDRF) of red snow algae (red; *n* = 70), green snow algae (green; *n* = 91), clean snow (blue; *n* = 38), and mineral dust-entrained snow (black; *n* = 36). Error band shows the 95% confidence interval. Vertical shaded bands show the position of Bands 4, 5, and 6 of WorldView 3's multispectral sensor.

As in Di Mauro et al. (2020) and Gray et al. (2020), chlorophyll absorbance features within reflectance factors were determined using the scaled integral method developed by Painter et al. (2001). Field-derived hyperspectral HDRF of red or green snow algae were convolved to the spectral response of WorldView 2 or 3, and the integral of chlorophyll a absorbance, I_*B*5_, calculated using WorldView 2 or 3-simulated “yellow,” “red,” and “red-edge” bands (Equation 1). Band positions for the WorldView 2 and 3 sensors are shown in Table 1, and the position of the bands used in Equation (1) relative to the HDRF of snow algae is shown in the Results section (Figure 3). Since WorldView 2 and 3 have different spectral response functions, sensor-specific I_*B*5_ equations were used to analyze their respective imagery.

(1) $$I_{B5}=\;\frac{R_{B4}(\lambda_{B6}-\lambda_{B5})+R_{B6}(\lambda_{B5}-\lambda_{B4})}{\lambda_{B6}-\lambda_{B4}}-R_{B5}/\frac{R_{B4}(\lambda_{B6}-\lambda_{B5})+R_{B6}(\lambda_{B5}-\lambda_{B4})}{\lambda_{B6}-\lambda_{B4}}$$

where I_*B*5_ is the scaled integral of Band 5, *R*_*Bn*_ is the HDRF for Band “*n*”, and λ_*Bn*_ is the wavelength at the center point of Band “*n*” (see Table 1). Relative to the use of Sentinel 2 in Gray et al. (2020), where analysis was limited to green snow algae, WorldView's yellow band at 605 nm corresponds to the lower shoulder of the chlorophyll a absorption feature, without interference from astaxanthin absorption. This meant that Equation (1) could be used upon WorldView multispectral imagery to detect both red and green snow algae.

**Table 1:** WorldView 2 and 3 band positions.

**Band**	**WorldView 2 center**	**WorldView 2**	**WorldView 3 center**	**WorldView 3**
	**wavelength (nm)**	**bandwidth (nm)**	**wavelength (nm)**	**bandwidth (nm)**
Coastal blue (B1)	447	62	426	57
Blue (B2)	478	73	481	72
Green (B3)	546	80	547	79
Yellow (B4)	608	48	605	49
Red (B5)	659	70	661	70
Red edge (B6)	724	50	724	51
NIR1 (B7)	833	136	832	134
NIR2 (B8)	949	187	948	182

*The lower and upper band edges and center wavelengths for the VNIR sensors on board WorldView 2 and 3 VNIR multispectral satellites*.

### 2.2. Remote Sensing

WorldView imagery over the Ryder Bay area from February 6, 2017 (WorldView 3), February 28, 2017 (Worldview 2), November 23, 2017 (Worldview 2), and December 28, 2017 (Worldview 2) was accessed through the DigitalGlobe Foundation. Imagery was processed and analyzed at 1.24 m ground sampling distance for Worldview 3 and 1.84 m for WorldView 2. Imagery was converted to surface reflectance using the Atmospheric and Radiometric Correction of Satellite Imagery (ARCSI) command line tool (Bunting and Clewley, 2019), which uses the 6S radiative transfer model (Vermote et al., 1997) to perform atmospheric correction. The 8m Reference Elevation Model of Antarctica (REMA) (Howat et al., 2019) was used to provide elevation data for atmospheric correction. Aerosol optical depth (AOD) data, collected on clear sky days by Rothera's POM 01 (Prede Co. Ltd) automatic sunphotometer and a handheld Microtops II sunphotometer (Solar Light), were used to provide values of AOD for the 6S atmospheric model. No corresponding AOD measurements were available for the 2017 Ryder Bay images, so average clear sky values recorded during the 2018 Ryder Bay field season were used. For the 2019 King George Island image, AOD was derived from an average of triplicate Microtops measurements, taken at the same time as the WorldView 3 image. Maritime aerosol profile and sub-arctic winter atmospheric profiles were used.

Each scene was masked using the filter functions presented in Gray et al. (2020). These remove false positives that return high I_*B*5_ values but correspond to saturated, noisy or mixed pixels, or other terrestrial vegetation. A spectral angle mapper (SAM) classification was performed in ENVI (Version 5.5 Exelis Visual Information Solutions, Boulder, Colorado) on the masked multispectral imagery to differenciate between green, red, dirty and clean snow. For this classification model, reference end-member spectra of red (*n* = 57) and green (*n* = 91) snow algae, mineral entrained snow (*n* = 36), and control plots of algae-free snow (*n* = 38) were derived from averaged HDRF, recorded using field spectroscopy (spectra of these end members are shown in Figure 3 in the Results section).

**Figure 4:**
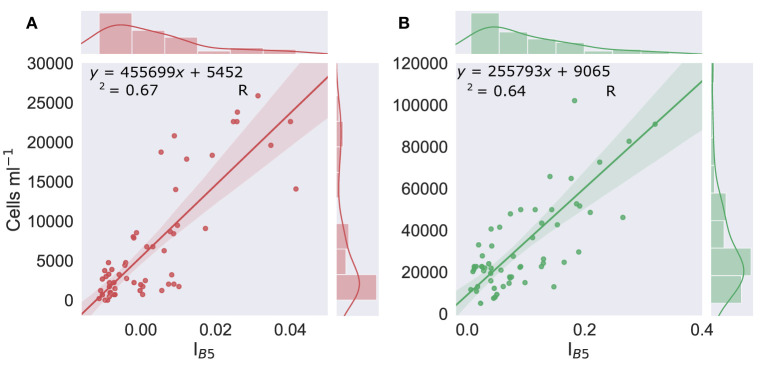
Cell density vs. I_*B*5_. Linear regression of the scaled integral of WorldView 3's Band 5 relative to Bands 4 and 6 (I_*B*5_) (derived from resampled field HDRF), vs. concentrations of red (left, graph **A**) (*n* = 57) or green (right, graph **B**) (n = 60) algal cells within the snow. Shaded area shows the 95% confidence interval. Marginal histograms show data distribution.

Chlorophyll absorbance was then mapped by applying I_*B*5_ (Equation 1) to each masked image. To relate this mapped absorbance to cell density, we used the *in situ* data collected with the field spectrometer and microscope. The lines of least squares regression of field spectroscopy-derived I_*B*5_ vs. measured cell density, shown here in Figure 4, were used to relate the I_*B*5_ value of each pixel to a cell concentration. For pixels identified as red snow by the SAM classification, cell density was calculated using the linear regression of red snow algae vs. I_*B*5_ (Figure 4A; Equation 3), whereas, for pixels classified as green snow algae, cell density was calculated using the linear relationship between green algal cell density and I_*B*5_ (Figure 4B; Equation 4). Single isolated pixels were discarded from analysis as they predominantly corresponded to sun glint from rock or heavily crevassed ice. Pixels returning a lower cell density than the intercept of Equations (3) or (4) were also discarded. As in Gray et al. (2020), we estimated snow algal biomass (dry mass) using averaged *in situ* measurements of red or green bloom layer thickness and snow density, and the pixel area, calculated based on mean row and column pixel dimensions (which vary according to the cross and in track viewing angle to normalize per pixel cell concentrations by volume). Average measured dry mass of a single red or green snow alga was then used to calculate dry biomass per pixel, with the mass of carbon present determined using the average measured %C in red and green blooms. All of these data are presented in Table 2.

**Table 2:** Snow algae *in situ* data.

	**Green *in situ***	**Red *in situ***	**Green WorldView 28/02/17**	**Red WorldView 28/02/17**
Snow algae cells ml^−1^ snow melt	1.9 × 10^5^ ± 3.8 × 10^5^ (*n* = 66)	3.4 × 10^4^ ± 1.0 × 10^5^ (*n* = 108)	7.3 × 10^4^ ± 3.0 × 10^4^ (*n* = 15,614)	3.3 × 10^4^ ± 1.5 × 10^4^ (*n* = 23,956)
Snow algae cells m^−2^ snow surface	2.9 × 10^9^ ± 3.8 × 10^9^ (*n* = 66)	8.0 × 10^8^ ± 3.4 × 10^9^ (*n* = 136)	3.9 × 10^8^ ± 1.6 × 10^8^ (*n* = 15,814)	3.1 × 10^7^ ± 1.4 × 10^7^ (*n* = 23,956)
Snow algae community dry mass (g m^−2^)	29.5 ± 29.9 (*n* = 50)	0.83 ± 1.4	9.1 ± 3.8 (*n* = 15,814)	1.9 ± 0.9 (*n* = 23,956)
Snow algal single cell mass (g)	2.4 × 10^−8^ ± 2.2 × 10^−8^ (*n* = 90)	6.2 × 10^−8^ ± 1.2 × 10^−7^ (*n* = 57)	–	–
Snow density (ml melt cc^−1^ snow)	0.58 ± 0.17 (*n* = 90)	0.49 ± 0.13 (*n* = 57)	–	–
Snow algal layer thickness (mm)	9.1 ± 5.9 (*n* = 90)	1.9 ± 3.3 (*n* = 57)	–	–
Snow algal %C	36.1 ± 9.5 (*n* = 82)	34.9 ± 10.3 (*n* = 17)	–	–

*Cell counts, snow physical properties, and carbon data of Antarctic green and red snow algae compared to remote-sensed estimates from Anchorage Island using WorldView 2 on February 28, 2017. Reported values are mean values (±1 standard deviation) from a combination of field work conducted in the Ryder Bay area of Adelaide Island (2018) and the Fildes Peninsula area of King George Island (2019). In situ data for green snow algae is also reported in Gray et al. (2020)*.

To compare with other terrestrial vegetation, the normalized difference vegetation index (NDVI), a proxy for detecting vegetation in multispectral imagery, was calculated using the image from February 28, 2017. This was done with Equation (2), utilizing the NDVI-2 band combination tested by Jawak et al. (2019) on Antarctic vegetation. We chose a lower NDVI threshold of 0.1 after Fretwell et al. (2011), as values less than this introduced significant noise into our analysis, where isolated pixels on targets known to not contain vegetation had NDVI values between 0.05 and 0.1. Pixels identified as snow algae that also had NDVI > 0.1 were clipped out of the NDVI map to allow comparison between snow algae and terrestrial vegetation.

(2) $$\begin{array}{l} NDVI=\frac{NIR\;2-Red}{NIR\;2+Red} \end{array}$$

### 2.3. Accuracy Assessment

**Figure 5:**
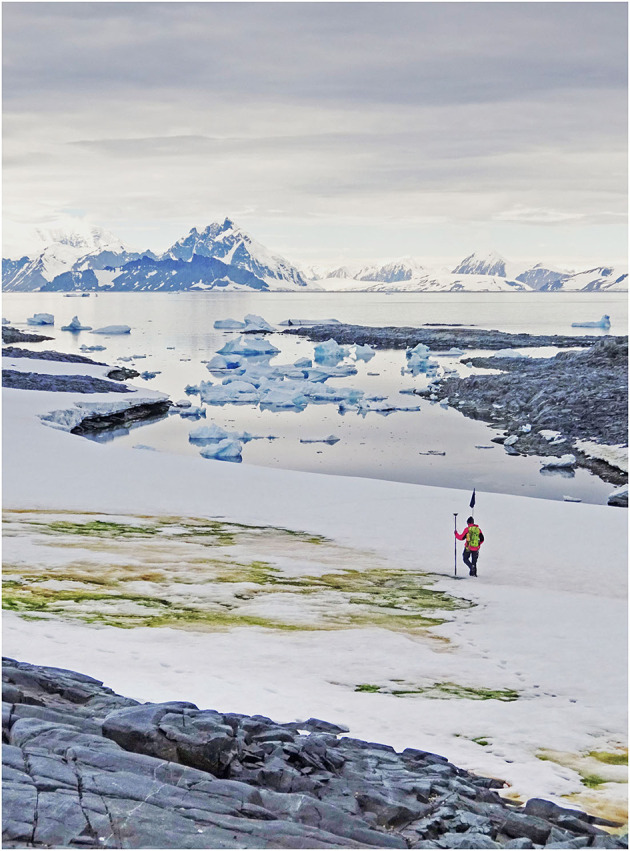
Snow algae blooms on Anchorage Island. Photograph of Andrew Gray GPS tracing a green snow algal bloom on south-western tip of Anchorage Island, January 31, 2018 (Credit: M. Davey).

During the 2018 and 2019 field seasons, locations where algae were visible on the surface of the snow pack were traced using a Trimble 5700 GPS receiver and Zepher Antenna (Ryder Bay; see Figure 5 for a photograph of GPS tracing), and an Emlid RS+ GNSS receiver (King George Island). Triplicate samples of green, red, and clean snow were collected from each bloom location and analyzed for cell density by light microscopy using the methodology described in Gray et al. (2020). GPS positions of field-checked blooms are shown in Figure 1. In the case of King George Island (Figure 1A), points were recorded within 1 day of the WorldView 3 image shown. GPS data of bloom locations in the Ryder Bay area were recorded in January and February 2018 (imagery from 2017). Accuracy of both the SAM classification and the location of snow algae identified using I_*B*5_ was assessed by calculating the Kappa coefficient of agreement using these ground-checked locations. For Ryder Bay images, where there was a significant time difference between ground observations and images, manual checking of true color imagery was also employed to corroborate Kappa scores. For the Ryder Bay images, 76 GPS referenced blooms were used to validate the images from February. Since we had near overlapping field observations for the Ryder Bay image from the December 28, 2017, 26 blooms locations recorded in early January 2018 were used to validate this image. For the King George Island image on February 1, 2019, 40 bloom locations that were recorded within 4 days of these dates were used for validation.

## 3. Results

### 3.1. *In situ* Sampling

#### 3.1.1. Bloom Phenology

Blooms in the Ryder Bay area, including those on Anchorage Island, were initially (early January 2018) observed as a green layer within the snowpack. Small patches of red snow were also noted on the surface of the snowpack in places in early January. Later in the season (early February), red and green snow algal blooms covered a visibly larger area. By mid-February, overlying snow had melted to expose the green algal layers, often on the surface of a dense, multi-year snowpack as shown in Figure 5. Pit sampling revealed green layers turning red in some instances (Figure 2C), although red blooms mostly appeared on the top of fresh snow, with no green cells detected within the underlying snowpack (Figure 2B). Several patches of mixed red and green snow were also evident, though it was unclear whether this was representative of different stages of life cycle or two distinct populations mixed through snow-melt action.

**Figure 6:**
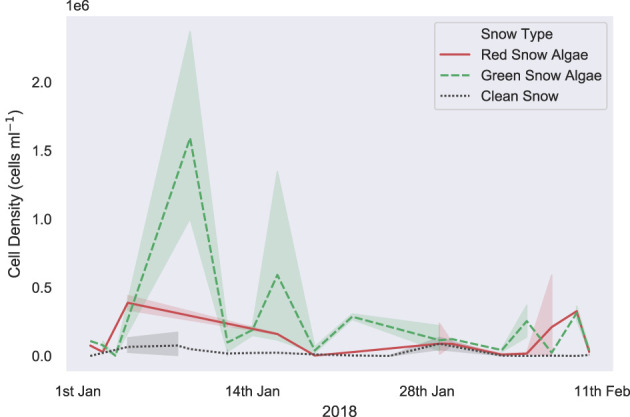
*In situ* snow algal cell density. Measured *in situ* algal cell density within clean, visibly red or visibly green snow, sampled for life cycle analysis throughout the 2017/2018 growth season in the Ryder Bay area, Antarctica. Samples are from Rothera Point, Anchorage Island, Lagoon Island, and Léonie Island. Shaded area shows one standard deviation.

**Figure 7:**
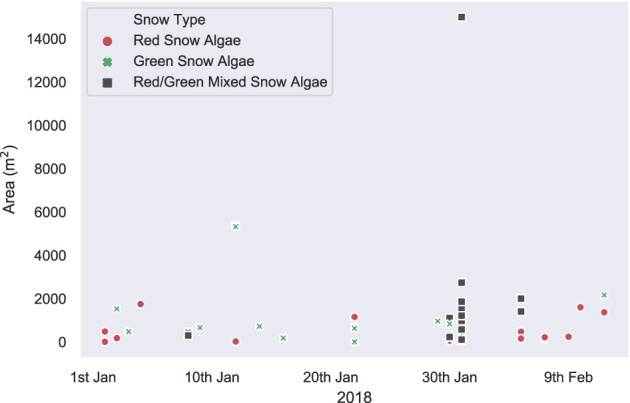
*In situ* bloom area measurements. *In situ* measurements of visible bloom area, recorded with a Trimble dGPS throughout the 2018 field season in the Ryder Bay area. Blooms recorded are from Rothera Point, Anchorage Island, Lagoon Island, and Léonie Island.

Figure 6 shows the algal cell density of visibly red, green, or clean snow throughout the 2017/2018 growth season. No significant trends were observed across the 42 days of observation. Average cell density (shown in Table 2) was 1.9 × 10^5^ cells ml^−1^ for green and 3.4 × 10^4^ for red blooms, with considerable variation observed between different blooms of the same color (respective standard deviations of 3.8 × 10^5^ and 1.0 × 10^5^). There were also no changes in measured visible bloom area through January and February during our observations in 2018 (Figure 7). Average bloom area was 1,110 ± 674 m^2^ for green blooms and 531 ± 364 m^2^ for red blooms. The cell densities measured within strongly colored patches of snow in 2018 were consistent with those recorded for Ryder Bay snow algae in 2015 (Davey et al., 2019), and are similar to cell densities recorded elsewhere in Antarctica (Hodson et al., 2017).

#### 3.1.2. Field Spectroscopy

Average HDRF of red and green snow algae, and clean and mineral entrained snow are shown in Figure 3. Both red and green algae exhibited strong absorption features at 680 nm from chlorophyll a, which were not present within the recorded spectra of clean or mineral dust-containing snow. Green snow algae HDRF also had strong absorption from chlorophyll b, a broad peak between c. 400 and 500 nm, which was present but less pronounced in red bloom spectra. Red blooms also showed absorption from carotenoids, such as astaxanthin, which when combined with chlorophyll b creates a broad flat absorption feature below 570 nm. Field HDRF indicate similar pigmentation to that observed through HPLC analysis of snow algae sampled from the same area (Davey et al., 2019) and are similar to red and green snow algae spectra reported elsewhere (Painter et al., 2001; Holzinger et al., 2016; Huovinen et al., 2018; Khan et al., 2021).

There was significant variation in the magnitude of HDRF within the visible/near infra-red (VNIR) region, as our sampling aimed to survey a wide range of cell densities to avoid extrapolation when applying our regression model to remotely sensed images. Lower cell concentrations, where coloration was just visible on the surface of the snow, typically had higher overall reflectance and a smaller chlorophyll absorption features at 680 nm. Where algae completely covered the snow surface, HDRF values were much lower within the VNIR range but also exhibited deeper chlorophyll a absorbance features. The relationship between cell density and the continuum-scaled integral of the chlorophyll a absorbance feature is shown in Figure 4. The linear relationship between cell density and I_*B*5_ (Figure 4) was high and significant for both red and green blooms, with respective Pearson's correlation coefficients of *r*(58) = 0.82, *p* < 0.01 and *r*(55) = 0.80, *p* < 0.01. R^2^ was, respectively, 0.67 and 0.64, and the standard error 4.3 × 10^4^ and 2.5 × 10^4^ cells ml^−1^. The lines of best fit, used to estimate cell density within a WorldView pixel, are shown in Equations (3) and (4), for red and green snow algae, respectively.

(3) $$\begin{array}{l} Red\;Cells\;ml^{-1}=\left(I_{B5}\times 455699\right)+5452 \end{array}$$

(4) $$\begin{array}{l} Green\;Cells\;ml^{-1}=\left(I_{B5}\times 255793\right)+9065 \end{array}$$

Likely causes of variation in these regression models include factors that affect the HDRF lineshape, for example, debris, mixed communities of red and green algal cells, and snow morphology, such as crystal structure and liquid water content (Cook et al., 2017). Variation may also arise from sampling geometry, with a fixed nadir viewing angle, but with aspect, slope angle, and the solar zenith varying between sampling sites. The y-intercepts of Equations (3) and (4) (the lines of best fit from Figures 4A,B) determined the lower limit of cell density detection within a WorldView pixel using this approach: 5.5 × 10^3^ cells ml^−1^ for red snow algae, and 9.1 × 10^3^ cells ml^−1^ for green snow algae. This is, respectively, one and two orders of magnitude smaller than our averaged measured cell densities (Table 2).

### 3.2. Remote Sensing Snow Algae

#### 3.2.1. Accuracy Assessment and Bias

**Figure 8:**
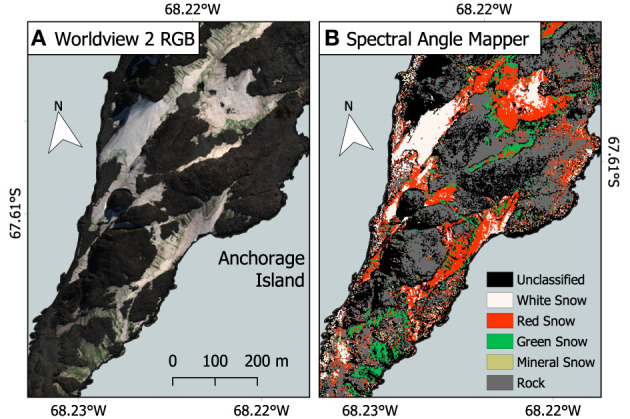
Spectral angle mapper (SAM) classification example. **(A)** Red–green–blue (RGB) true color WorldView 2 image of a section of Anchorage Island from February 28, 2017. Image extent is highlighted in Figure 1. **(B)** Output from the spectral angle mapper classification (SAM), used to determine which pixels contained red, and green snow algae for later calculation of pixel cell density.

Figure 8 shows the output from the SAM classification. Run only on snow within a scene, SAM performed well in classifying between red or green snow algae, white and mineral-entrained snow, with Kappa scores of 0.68 on December 28, 2017, 0.81 on February 6, 2017, and 0.64 on February 28, 2017, when compared to ground GPS data from the subsequent season. The November 23, 2017 image differed greatly from our ground GPS data, with seasonal snow covering most of the islands. Instead, SAM output was assessed by manual checking against true color imagery, where it correctly classified most clean snow but did not perform well with areas of shadow, misclassifying them as mineral-laden or red algal blooms. SAM misclassification in all images predominantly was related to red snow being assigned as mineral entrained snow or clean snow, or very dark patches of green snow algae (which may be classified as rock/terrestrial vegetation within our SAM end member framework). We were unable to classify or separate red or green snow in the image of King George Island, as its blue and green bands were saturated over snow.

**Figure 9:**
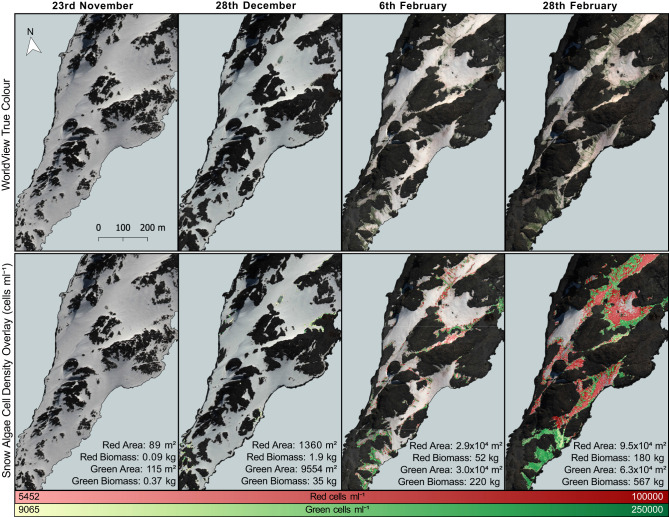
Red and green snow algal distribution and modeled cell density. Area coverage and (dry) biomass estimates are given for red and green snow algae on Anchorage Island. All images are from 2017. Map extent is shown in Figure 1B.

Figure 9 shows the output of our approach to identifying snow algae within WorldView 2 and 3 images, combining filter masking, SAM, I_*B*5_, and Equations (4) or (3). Since it was our only WorldView image with concurrent field measurements, we performed an accuracy assessment using georeferenced blooms on the saturated image of King George Island. All GPS-logged patches of snow algae were correctly identified by our model; however, its Kappa score was low (0.59) as we were unable to accurately differentiate algae from mineral-laden snow, with SAM classification performing poorly on the saturated image. For Ryder Bay images, Kappa scores of 0.79 on December 28, 2017, 0.59 on February 6, 2017, and 0.82 on February 28, 2017 were observed. As with SAM validation, November 23, 2017 scene was assessed by manually checking against true color imagery. Areas with visible green/orange coloration to the snow were classified as snow algae, as were patches close to Rothera Station that appeared white in this image, but had developed into green blooms by December 28, 2017, possibly indicating sub-surface blooms.

At center swath and on flat ground, WorldView 3 has a ground sampling distance of 1.24 m for its VNIR bands, with WorldView 2 only slightly larger at 1.84 m. This allows detection of smaller blooms than is possible using Sentinel 2 or Landsat imagery, yet our field observations indicate that blooms were still patchy and heterogeneous at this scale. Any chlorophyll absorbance from the presence of snow algae will be integrated across a pixel according to its point spread function (Radoux et al., 2016) with a theoretical lower limit of detection for bloom area being based upon the bloom's cell density and whether it crosses through the center or is positioned at the border of a pixel. Based upon average *in situ* cell densities from Table 2, the minimum detectable cell densities from Equations (4) and (3), and assuming a bloom crosses through the center of a pixel otherwise containing clean snow, we estimate the minimum detectable bloom area to be 0.09 and 0.06 m^2^ for green blooms, and 0.3 and 0.2 m^2^ for red blooms, in WorldView 2 and 3 imagery, respectively. Mixed pixels containing rock or vegetation alongside green snow algae would likely be excluded from the study based on the filter functions applied before analysis (Gray et al., 2020).

Spatial biases of remote-sensed snow algal area estimates may be caused by misclassification of pixels, subpixel blooms being counted as one pixel in area, blooms growing under the surface layer of snow being excluded from observation, and error associated with pixel dimensions on the snow surface. Each of these will cause over- or underestimation of area and will affect red and green blooms differently throughout the growth season. Misclassification likely led to underestimation of red bloom area, where pixels containing red snow algae were incorrectly labeled as mineral entrained or clean white snow by the SAM classifier. Conversely, misclassification likely caused overestimation of green bloom area, as some noisy or dark pixels with high I_*B*5_ remained after filtering and were often misclassified as green snow algae by our SAM classifier. These were typically on angular rocky structures or crevassed ice and required manual removal prior to analysis. Overestimation of the area of subpixel blooms will likely cause greater bias at the beginning of the season, where the exposed area of blooms may be smaller. However, this is also when we expect to have greater underestimation of bloom area, as overlying snow obscures growth from view. The latter will mostly affect green blooms, as red blooms in coastal Antarctica have predominantly been observed to inhabit the surface of snow (Remias et al., 2013; Davey et al., 2019). Spatial bias resulting from error in pixel dimension introduces uncertainty when comparing different images/sensors, as the off-nadir and azimuth viewing angles with respect to surface topography will result in different pixel area errors for the same blooms within different images. The areas reported here are given minimum and maximum range bounds based upon potential bias from misclassification (based on that image's accuracy assessment) and the range of pixel area within a scene (based on image-specific viewing geometry only). Errors associated with foreshortening and elongation of pixels because of satellite viewing geometry were not considered in this paper and should be considered in greater depth in future study.

Assuming pixels contain only red or green snow, and do not contain mixed populations, error for remote-sensed cell density estimates is largely captured by the standard error of our regression models relating I_*B*5_ to cell density (Figure 4). This standard error would relate to errors in field spectroscopy-based I_*B*5_ measurements, the associated cell density measurement and non-snow algae factors that may influence lineshape and hence I_*B*5_. These may be factors, such as mineral dust, snow crystal morphology, and bi-directional reflectance factor (BRDF) effects. Biomass estimates have greater uncertainty because they are parameterized using averaged field measurements rather than mapped or remote sensing-derived snow density, thickness, or cellular mass. Biomass estimates are given with maximum and minimum values based on propagated error from pixel area, the standard error of red or green snow algae regression models, and each empirically derived parameter used for calculation. Error is more complex where pixels contain mixed red and green populations, as SAM may classify a pixel as red or green based upon the greater number of red or green cells in that pixel rather than by greatest area coverage, although the position of red or green cells within a pixel relative to its point spread function will also influence classification. In this case, red snow algae in a pixel classified as green will be enumerated based upon the relationship between I_*B*5_ and green algae (Equation 4) rather than I_*B*5_ and red algae (Equation 3) and vice versa. Dry biomass estimates will similarly be based upon incorrect field measurements. Future work should address this uncertainty, through spectral unmixing approaches, or analysis of higher resolution drone imagery.

#### 3.2.2. WorldView Identification of Snow Algae on Anchorage Island

**Figure 10:**
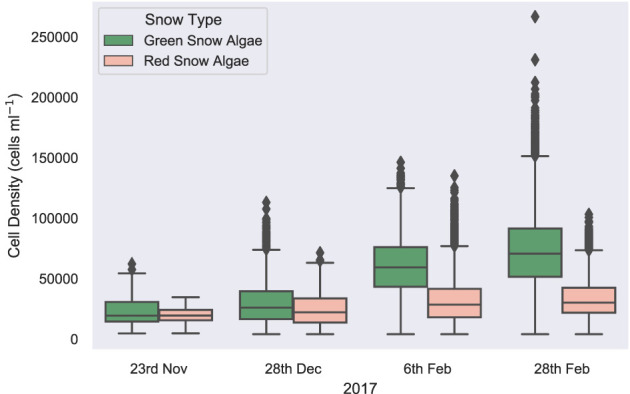
Remotely sensed cell density. A boxplot showing the change in average snow algal cell density for Anchorage Island, recorded by WorldView 2 and 3 imagery, through a growing season.

Remote sensing results reported here focus on Anchorage Island alone, as the footprint of each WorldView image of Ryder Bay was different, with sections of Léonie Island cropped from several images. Figure 9 maps the change in distribution of snow algal biomass and area over the southwestern tip of Anchorage Island on February 6, February 28, November 23, and December 28. To complement this map, Figure 10 shows the change in remote-sensed estimates of snow algal cell density across these time points. Although the images are from the same year, rather than the same growth season, our analysis is presented as a seasonal progression to explore changes from early (November/December) to late (February) season bloom development.

On November 23, 2017, green snow algae were identified at several locations where sea ice had thinned close to the shore and green or red/orange coloration appeared at the snow surface. These covered a small total area, with pixels identified as green snow algae covering 115 m^2^ (min: 114 m^2^; max: 118 m^2^) and pixels identified as red snow algae covering 89 m^2^ (min: 88 m^2^; max: 91 m^2^). These blooms were not densely populated, with a green pixel average cell density of 2.4 × 10^4^ cells ml^−1^ ± 1.1 × 10^4^ (*n* = 26) and a red pixel average cell density of 2.0 × 10^4^ cells ml^−1^ ± 1.3 × 10^4^ (*n* = 20). Dry biomass was, respectively, 0.37 kg (min: 6 g; max: 1.5 kg) and 0.09 kg (min: 38 g; max: 1.0 kg) for green and red blooms. By December 28, 2017, green snow algae were identified at the edges of snow packs and in troughs in the landscape. Both area (mean: 9.6 × 10^3^ m^2^; min: 8.7 × 10^3^ m^2^; max: 9.7 × 10^3^ m^2^) and average pixel cell density (3.0 × 10^4^ cells ml^−1^ ± 1.3 × 10^4^; *n* = 1749) had increased relative to the image from November 23, 2017, with dry biomass now totaling 42 kg (min: 0.6 kg; max: 179 kg) for Anchorage Island. Red snow algae were identified alongside these green patches, though they covered a smaller area (mean: 1.4 × 10^3^ m^2^; min: 1.2 × 10^3^ m^2^; max: 1.4 × 10^3^ m^2^). Here, red pixels averaged 2.5 × 10^4^ cells ml^−1^ ± 1.5 × 10^4^ (*n* = 249) and contained an estimated 1.9 kg (min: 0.7 kg; max: 20.3 kg) of dry biomass. By February 6, 2017, the area of visible green blooms had increased to 3.02 × 10^4^ m^2^ (min: 2.36 × 10^4^ m^2^; max: 3.20 × 10^4^ m^2^), as had the area of red snow algae, which was now similar to that of green (2.89 × 10^4^ m^2^; min: 2.10 × 10^4^ m^2^; max: 2.91 × 10^4^ m^2^). Average cell density for both red and green blooms had increased relative to December 28 image (5.0 × 10^4^ cells ml^−1^ ± 1.7 × 10^4^; *n* = 12,094 and 4.1 × 10^4^ cells ml^−1^ ± 2.3 × 10^4^ (*n* = 11,561), respectively), green blooms significantly so (*t*-test: *t* = 67; *P* ≤ 0.01). Total dry biomass for Anchorage Island within this image was 52 kg (min: 15 kg; max: 537 kg) for red snow algae and 224 kg (min: 2.8 kg; max: 979 kg) for green snow algae. By February 28, 2017, red snow algae covered a greater area than green, averaging 9.48 × 10^4^ m^2^ vs. 6.26 × 10^4^ m^2^ (respective minimum area: 8.65 × 10^4^ m^2^ and 5.56 × 10^4^ m^2^; respective maximum area: 9.49 × 10^4^ m^2^ and 6.27 × 10^4^ m^2^). Green cell density had significantly increased (*t*-test: *t* = 41; *P* ≤ 0.01) relative to February 6, 2017, averaging 6.0 × 10^4^ cells ml^−1^ ± 2.2 × 10^4^ (*n* = 15,814) in February 28, 2017 image, while red bloom cell densities remained similar within both February images (4.3 × 10^4^ cells ml^−1^ ± 2.0 × 10^4^; *n* = 23,956). Dry biomass on February 28, 2017 was 567 kg (min: 8 kg; max: 2,340 kg) for green blooms and 180 kg (min: 34 kg; max: 1,850 kg) for red blooms. The entirety of the WorldView 2 image from the February 28, 2017 (extent is shown in Figure 1B) contained 1.1 × 10^5^ m^2^ (min: 9.9 × 10^4^ m^2^; max:1.1 × 10^5^ m^2^) of green snow algae and 2.6 × 10^5^ m^2^ (min: 2.3 × 10^5^ m^2^; max: 2.6 × 10^5^ m^2^) of red snow algae, respectively, contributing 778 kg (min: 11 kg; max: 3220 kg) and 388 kg (min: 137 kg: max: 3,980 kg) of dry biomass to the area.

Red blooms had closer agreement between remote-sensed and *in situ* measurements of cell density, relative to green ones, with derived estimates, respectively, averaging 97 and 38% of *in situ* cell counts. It is likely this discrepancy is partially because the *in situ* mean overestimates average green bloom cell density due to sampling bias when in the field. Green blooms form dense mats of algae on relatively small patches of snow, whereas red blooms were typically more evenly distributed across a broader area, thus are less subject to bias when sampling. Integrating small dense patches of green algae over the area of one pixel may cause remote-sensed estimates of green algal cell density to underestimate real-world values. This spreading effect is also more likely to influence green blooms than red.

Based on dGPS data from two surveys on Anchorage Island, conducted on January 31 and February 12, 2018, blooms ranged from 60 to 2,739 m^2^ in size, with the average bloom area being 962 m^2^. Remotely sensed area estimates of discrete blooms from February 6, 2017 suggests the average bloom size was 12 m^2^, with the maximum observed bloom being 1,035 m^2^. At this point, Anchorage Island had 3,277 discrete blooms of snow algae with blooms under 100 m^2^ contributing to 60% of total snow algal area. Blooms appear smaller within the satellite imagery because of differences by which blooms were separated into discrete entities. The remote sensing analysis rigidly separates blooms that do not intersect, whereas field dGPS surveys aggregate adjacent patches (as shown in Figure 5) where it was evident that subsurface algae connected blooms that were visible on the surface.

#### 3.2.3. Snow Algae vs. Other Terrestrial Vegetation

**Figure 11:**
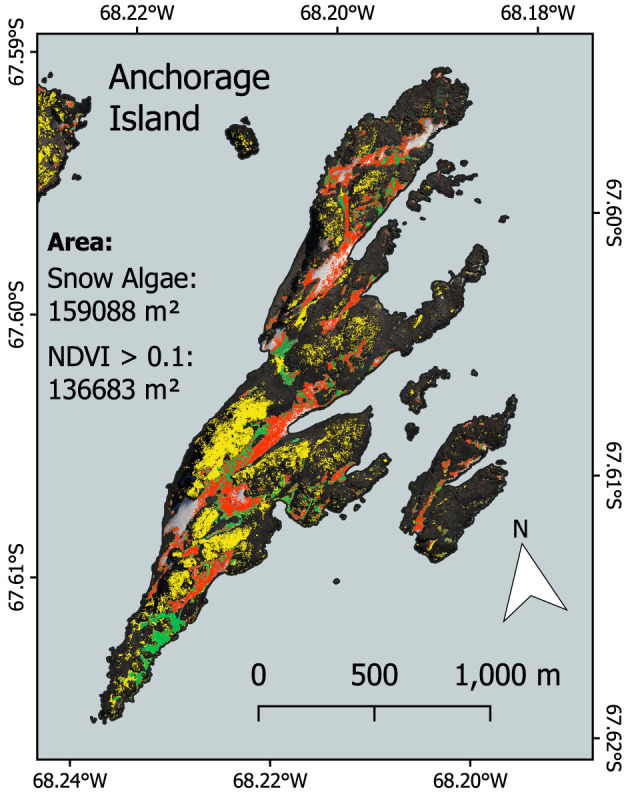
Comparison between snow algal area and normalized difference vegetation index (NDVI) (a vegetation proxy) for Anchorage Island. Red and green pixels show the area covered by red and green snow algae, respectively. Yellow indicates pixels with an NDVI > 0.1, which indicates other vegetation (moss and lichen).

Figure 11 shows snow algae alongside pixels with an NDVI > 0.1 on Anchorage Island on February 28, 2017. This image had the largest area coverage of snow algae, and the lowest area coverage of snow, meaning seasonally snow-covered terrestrial vegetation is likely to be exposed. For Anchorage Island, the total area covered by snow algae was 1.57 × 10^5^ vs. 1.37 × 10^5^ m^2^ for pixels with an NDVI > 0.1. The average NDVI for pixels identified as vegetation (NDVI > 0.1) on Anchorage Island was 0.13 ± 0.07. Of 15 GPS-logged locations where moss or lichen covered an area larger than 1 m^2^, recorded in February 2018, 80% had NDVI values > 0.1.

## 4. Discussion

### 4.1. WorldView Satellites to Map Snow Algae

We have used WorldView 2 and 3 multispectral imagery to map changes in Antarctic snow algae during the summer growth season. The high spatial resolution of WorldView satellites has significantly improved upon previous satellite-based maps of snow algae, producing detailed area coverage and cell density estimates that let us explore the seasonal development of snow algal blooms. Additionally, WorldView's high spectral resolution meant we were able to map red algal blooms alongside green ones, addressing a major limitation of our previous study (Gray et al., 2020). Two ground validation campaigns (in 2018 and 2019) monitored the seasonal development of snow algae in Antarctica and collected field spectra of snow algae. These data were then used to develop a model to detect and estimate algal cell density in snow, by using the scaled integral of a chlorophyll absorption feature evident within both field reflectance factors and satellite pixels. Positional data were also collected to assess accuracy when applying this approach to WorldView imagery. Using this method, we successfully identified red and green snow algae on Anchorage Island, Antarctica, estimating peak coverage to be 9.48 × 10^4^ and 6.26 × 10^4^ m^2^, respectively. Despite covering a smaller area, green snow algae had greater cell density than red blooms (6.0 × 10^4^ and 4.3 × 10^4^ cells ml^−1^, respectively), and had higher average layer thickness (see Table 2). This meant that green algae contributed more overall to terrestrial biomass on the island than red algae (567 vs. 180 kg).

**Figure 12:**
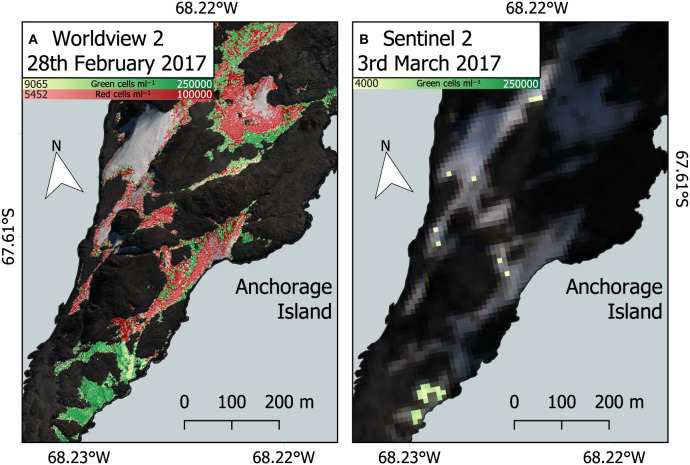
Comparison between WorldView 2 and Sentinel 2. **(A)** WorldView 2 analysis of red and green snow algal distribution and cell density on February 28, 2017, Anchorage Island, Antarctica. **(B)** Green snow algal distribution and cell density based on a Sentinel 2 image from March 3, 2017, using methodology described by Gray et al. (2020). Note cloud shadow is partially obscuring the north east section in this image.

Our work builds upon previous use of Sentinel 2 imagery to study snow algae in Antarctica (Huovinen et al., 2018; Gray et al., 2020; Khan et al., 2020, 2021) and shows the advantage gained by using high spatial and spectral resolution satellites, such as WorldView 2 or 3. Figure 12 compares snow algae identified through the methodology presented here, with that used in Gray et al. (2020), which used coarser-resolution Sentinel 2 satellite imagery. Figure 12B is a Sentinel 2 image that was captured 3 days after the WorldView 2 image in Figure 12A. Across the same area of Anchorage Island, Sentinel 2 was only able to detect 14.8% of the area of green snow algae identified by WorldView 2. Due to coarser spectral resolution, Sentinel 2 was unable to reliably detect red blooms, meaning that the total area of snow algae detected by Sentinel 2 was 5.7% of the combined area of red and green blooms detected with WorldView 2. WorldView 2 pixels cover a 25x smaller area than Sentinel 2 pixels, resulting in far fewer mixed pixels at the edges of blooms that would be filtered out when analyzing Sentinel 2 imagery. Smaller pixels also allows WorldView 2 to detect much smaller blooms [0.09 m^2^ vs. 11 m^2^ for Sentinel 2 (Gray et al., 2020)] with less chance of misclassification. The total bloom area for green snow algae on the Antarctic Peninsula detected using Sentinel 2 in Gray et al. (2020) was 1.9 km^2^. Since a large proportion of this area was related to several very large blooms, where the limitation of Sentinel 2's minimum area detection limit would be less problematic, we cannot simply scale the difference observed between satellites for Anchorage Island in Figure 12 to improve our Peninsula area estimate. Instead, it serves to highlight the importance of smaller blooms in contributing to the total area covered by snow algae. It also shows a need to refine methods for automatic sub-pixel bloom detection when using Sentinel 2 or similar imagery to study snow algae over large areas.

Of the methods employed here for identifying snow algae, most uncertainty relates to the SAM classifier as a method for discriminating between snow end members as “clean,” “mineral-laden,” “red,” or “green” snow algae. SAM was used over other classification methods for this study as it is relatively robust against BRDF effects (Weyermann et al., 2009). However, snow and ice have diverse and dynamic morphology, where grain size, density, and liquid water content may all change significantly throughout the day and influence the scattering of light (Dozier et al., 2009; Nolin, 2011). Moreover, our end member HDRF were collected over a narrow time period relative to the snow algal growth season, and so may not be representative of a full range of snow conditions. Spectral unmixing approaches have been used to separate mineral-laden snow from snow algae-containing snow with some success within Sentinel 2 images (Huovinen et al., 2018). However, unconstrained linear unmixing classification using the same end member spectra as used to populate our SAM classifier yielded poor performance on our WorldView imagery, and so was not considered further here. Indices methods for identifying mineral impurities within snow, such as those developed by Di Mauro et al. (2015) and Kokhanovsky et al. (2021), may improve the reliability of classifying red snow algae when combined with our SAM approach. Future work should focus on refining ensemble methods to improve classification of snow and its mineral and biological impurities over a wider range of seasonal conditions within multispectral satellite imagery.

For studying the ecology of snow algae, the synoptic view provided by satellites escapes the sampling bias of *in situ* observations while providing temporal characterization of fundamental properties, such as area and cell density across large areas. Remote sensing is not an alternative to field-based observations, however, as it misses the complexity of processes within the snow pack, both in terms of algal diversity and the physical stratification of algae within the snow itself. Moreover, coastal Antarctic snows are host to a variety of other microbial constituents that are not visible for remote detection, yet are important within biogeochemical processes, such as nutrient transfer and carbon cycling (Antony et al., 2016; Malard et al., 2019). Moreover, pixel size and processing routines used to identify snow algae introduce their own biases and are discussed here. The use of high spatial resolution WorldView imagery in this paper goes some way to addressing these biases to give a more complete picture of snow algae as an ecosystem relative to what has been achieved with the freely available Sentinel 2 imagery in Antarctica (Huovinen et al., 2018; Gray et al., 2020). Unfortunately, as this imagery needs to be tasked, and is relatively expensive, it is currently unsuitable for routine monitoring at large scales.

### 4.2. Snow Algal Seasonal Change and Phenology

The WorldView image of Anchorage Island from November 23, 2017 shows algae appearing where snow is overlying coastal inlets, where sea ice has persisted. With no *in situ* observations this early in the season, it is hard to confidently state whether these pixels are snow algae. The inlets where algae were identified by our model coincide with valleys further inland, meaning that early melt water could have been channeled here, or snow may be saturated from proximity to ocean water, causing wet conditions for algae to grow in the snowpack. Alternatively, we may be seeing a false positive from light diffracting through thin sea ice, or marine algae exposed from ice disturbance. Nonetheless, December's WorldView image mirrored our field observations, with small patches of green and red snow algae appearing at the melting edge of snow banks. Observations from snow pits dug in January at both Ryder Bay and King George Island field sites suggest that this green snow algal layer will extend up into the snowpack within a melt water-rich layer of snow/slush, sandwiched between overlying seasonal and compacted multi-year snow layers. Depending on the thickness and freshness (Perovich, 2007) of overlying snow, this layer of snow algae may be shielded from satellite observation, and hence our area estimates for December likely underestimate the actual extent of growth.

A significant change was observed in the late-season images, where green snow algae had appeared in the upper elevations of snow banks, exposed where the seasonal snow pack had melted away in runnels (see Figure 9). Field observations from Anchorage Island on January 31, 2018 indicated less extensive green snow algae coverage relative to February 6, 2017 WorldView image, though larger green patches were starting to appear (see Figure 5) in the same locations that were identified as containing snow algae by 2017 remote sensing imagery. They also followed the same pattern of exposure, melting out from underneath seasonal snow on the upper margins of snow fields. Average remote-sensed cell density estimates increased significantly between November 23 and February 28 images, suggesting considerable green algal growth between early and late season. We postulate that the maximum area coverage of green snow algae was achieved while buried underneath seasonal snow cover, where liquid water and nutrients are supplied by snow melt percolating through the algal layer (Nowak et al., 2018). This means that late-season remote sensing observations can be diagnostic of processes occurring earlier in the melt season, yet also highlights a primary limitation of optical remote sensing methods for snow alga (or algae) ecology: uncertainty relating to buried and obscured blooms.

Field observations indicated that red blooms primarily occurred on the surface of seasonal snow, sometimes appearing to bloom after fresh snow fall, although red coloration could also appear on underlying multi-year snow, presumably having been deposited by snow melt. The blooms on the seasonal snow surface surveyed on Anchorage Island on January 31 were red and/or orange in color (e.g., Figure 2B), and again were evident in similar locations to red snow algae identified in February 6, 2017 image. By February 28, 2017, red snow algae covered a greater area than green and, combined, snow algae covered 72% of the snowpack on Anchorage Island. Despite snow algal area reaching its observed maximum in February 28, 2017 image, there had also been significant loss of algae-inhabited snow by this point in the melt season. Between February 6 and 28, 1.2 × 10^4^ m^2^ of snow containing green algae, and 1.0 × 10^4^ m^2^ of snow containing red algae had melted out completely, and 13.8% of the total bloom area is identified in February 28, 2017 image.

Interestingly, we observed green snow algal blooms of similar sizes on the same snow banks across multiple growth seasons. Imagery from 2017 closely matches field observations from 2018, and indeed green coloration is visible on these same snow patches on the southern tip of Anchorage Island within 2020 Sentinel 2 imagery (data not shown). This may be an indication that the previous year's bloom seeds subsequent year's growth, where blooms on multi-year snow are buried by fresh snowfall (Hoham and Duval, 2001; Hoham and Remias, 2020). Figure 9 shows large red blooms forming adjacent to green. Along with observations of red cells forming on top of layers of green snow algae in several snow pits (see Figure 2C), this perhaps indicates that the red blooms on Anchorage Island are an encystment phase of an underlying green bloom. In some cases, this was confirmed, as snow that was red on February 6 was replaced with green snow by February 28, presumably as the cyst-containing seasonal snow melted out completely. Some in-field observations of red blooms, however, indicated that red snow algae were not associated with underlying vegetative cells, suggesting that aerial dispersal of cells onto the snowpack may also be an important factor influencing the colonization of snow (Marshall and Chalmers, 1997; Muller et al., 2001; Procházková et al., 2019). This may be possible to test with a remote-sensed phenology, such as in Figure 9, but we see no evidence of red blooms melting out to white snow in this case. Additionally, although there is increasing evidence that red cells have a bioalbedo advantage over green cells in seeding and establishing blooms (Dial et al., 2018), we do not have any systematic evidence in this study to support this and would require further investigation for such mixed green–orange–red color algal populations.

Our results indicate that 2017 snow algal coverage on Anchorage Island was greater in area than other terrestrial vegetation combined. Anchorage Island vegetation is predominantly mosses and lichens, with some *Prasiola* species also present (Bokhorst et al., 2007). Our results indicated that NDVI values on Anchorage Island were generally low (averaging 0.13), and consistent with those identified for lichen species in Landsat imagery by Casanovas et al. (2015). Casanovas et al. (2015) showed NDVI to perform relatively poorly when mapping Antarctic lichen species because of their low NDVI under dry conditions. However, though we did not perform species-specific ground validation for terrestrial vegetation, NDVI appeared to perform well, identifying large areas of lichen-covered rock toward the south of Anchorage Island. The results of Fretwell et al. (2011) and Casanovas et al. (2015) indicate that we may have missed some vegetation by choosing a 0.1 threshold for our NDVI value, in which case maximum snow algal area may be similar to that of terrestrial vegetation. We observed significant false positives when applying a lower 0.05 NDVI threshold on our February 28, 2017 image, however, and so cannot accurately use these data in our comparison. Future work may apply the full remote sensing framework devised by Jawak et al. (2019) for more robust identification of terrestrial vegetation in WorldView scenes to allow for better comparison between Antarctic vegetation types.

## 5. Conclusions

Our primary aim here was to improve remote sensing methods for detecting and monitoring snow algal blooms and to use them to assess fundamental aspects of the ecology of these organisms. We have made the first use of high-resolution WorldView 2 and 3 imagery from 2017 to identify blooms of red and green snow algae as they grow in the snow pack on Anchorage Island, Antarctica. From this imagery, we have produced estimates of cell density and area coverage, showing how red and green blooms developed separately as the melt season progressed. Together, red and green blooms grew to cover an area greater than other terrestrial vegetation on the island. This remote sensing study was underpinned by two field seasons in Antarctica, in 2018 and 2019, to build the empirical model which relates spectral reflectance to cell density and to spatially validate our model output.

WorldView imagery provided a significant improvement over previous efforts using Sentinel 2 imagery, with both red and green blooms automatically detected using the methodology developed for Sentinel 2 imagery by Gray et al. (2020). The higher resolution allows blooms to be picked out in far greater detail than previously achieved, meaning that blooms occurring on the edge of snow packs can be included in our analyses. Comparison of snow algal area estimates between WorldView and Sentinel 2 imagery implied that the total area of snow algae on the Antarctic Peninsula estimated in Gray et al. (2020) was a significant underestimate. However, until higher resolution multispectral imagery is routinely recorded over Antarctica, and is freely distributed, Sentinel 2 remains a more practical option for monitoring snow algae on large scales. Although there are still significant uncertainties associated with using satellites to monitor snow algae, high-resolution satellite imagery, such as WorldView offers the chance to perform relatively detailed analyses of blooms in specific areas through time, and to study how they are affected by factors, such as temperature and precipitation and how they, in turn, influence the albedo of the snow surface. Synoptic monitoring of snow algal growth and development during the Antarctic summer may yield further insights into their ecological strategies, metabolic load and life cycle, and how either may be affected by climatic changes in Antarctica.

## Data Availability Statement

The raw data supporting the conclusions of this article will be made available by the authors, without undue reservation.

## Author Contributions

MD, AG, LP, and PC designed and planned the fieldwork and logistics. AG, MD, and MM carried out the fieldwork. MD, MK, AG, AS, and MM planned and analyzed the field samples at Rothera Research Station, Escudero Base, and Cambridge. AG led the remote sensing, validation, and geospatial analysis with input from PF and MD. AG led the writing of the manuscript with all authors contributing and editing the text. All authors have seen and approved the final version.

## Conflict of Interest

The authors declare that the research was conducted in the absence of any commercial or financial relationships that could be construed as a potential conflict of interest.

## References

[B1] Antony R. Sanyal A. Kapse N. Dhakephalkar P. K. Thamban M. Nair S. (2016). Microbial communities associated with Antarctic snow pack and their biogeochemical implications. Microbiol. Res. 192, 192–202. 10.1016/j.micres.2016.07.004 27664737

[B2] Bidigare R. R. Ondrusek M. E. Kennicutt M. C. Iturriaga R. Harvey H. R. Hoham R. W.. (1993). Evidence a photoprotective for secondary carotenoids of snow algae. J. Phycol. 29, 427–434. 10.1111/j.1529-8817.1993.tb00143.x

[B3] Boetius A. Anesio A. M. Deming J. W. Mikucki J. A. Rapp J. Z. (2015). Microbial ecology of the cryosphere: sea ice and glacial habitats. Nat. Rev. Microbiol. 13, 1–14. 10.1038/nrmicro3522 26344407

[B4] Bokhorst S. Huiskes A. Convey P. Aerts R. (2007). The effect of environmental change on vascular plant and cryptogam communities from the Falkland Islands and the Maritime Antarctic. BMC Ecol. 7:15. 10.1186/1472-6785-7-15 18093288 PMC2234391

[B5] Bunting P. Clewley D. (2019). Atmospheric and Radiometric Correction of Satellite Imagery (ARCSI). Available online at: https://www.arcsi.remotesensing.info/ (accessed May 12, 2020).

[B6] Casanovas P. Black M. Fretwell P. Convey P. (2015). Mapping lichen distribution on the Antarctic Peninsula using remote sensing, lichen spectra and photographic documentation by citizen scientists. Polar Res. 34:25633. 10.3402/polar.v34.25633

[B7] Cook J. M. Hodson A. J. Gardner A. S. Flanner M. Tedstone A. J. Williamson C.. (2017). Quantifying bioalbedo: a new physically based model and discussion of empirical methods for characterising biological influence on ice and snow albedo. Cryosphere 11, 2611–2632. 10.5194/tc-11-2611-2017

[B8] Cubaynes H. C. Fretwell P. T. Bamford C. Gerrish L. Jackson J. A. (2019). Whales from space: four mysticete species described using new VHR satellite imagery. Mar. Mammal Sci. 35, 466–491. 10.1111/mms.12544

[B9] Davey M. P. Norman L. Sterk P. Huete-Ortega M. Bunbury F. Loh B. K. W.. (2019). Snow algae communities in Antarctica: metabolic and taxonomic composition. New Phytol. 222, 1242–1255. 10.1111/nph.15701 30667072 PMC6492300

[B10] Di Mauro B. Fava F. Ferrero L. Garzonio R. Baccolo G. Delmonte B.. (2015). Mineral dust impact on snow radiative properties in the European Alps combining ground, UAV, and satellite observations. J. Geophys. Res. Atmos. 120, 6080–6097. 10.1002/2015JD023287

[B11] Di Mauro B. Garzonio R. Baccolo G. Franzetti A. Pittino F. Leoni B.. (2020). Glacier algae foster ice-albedo feedback in the European Alps. Sci. Rep. 10:4739. 10.1038/s41598-020-61762-0 32179790 PMC7075879

[B12] Dial R. J. Ganey G. Q. Skiles S. M. (2018). What color should glacier algae be? An ecological role for red carbon in the cryosphere. FEMS Microbiol. Ecol. 94:fiy007. 10.1093/femsec/fiy007 29346532

[B13] Dierssen H. M. Smith R. C. Vernet M. (2002). Glacial meltwater dynamics in coastal waters west of the Antarctic peninsula. Proc. Natl. Acad. Sci. U.S.A. 99, 1790–1795. 10.1073/pnas.032206999 11830636 PMC122272

[B14] Dozier J. Green R. O. Nolin A. W. Painter T. H. (2009). Interpretation of snow properties from imaging spectrometry. Rem. Sens. Environ. 113, S25–S37. 10.1016/j.rse.2007.07.029

[B15] Fretwell P. T. Convey P. Fleming A. H. Peat H. J. Hughes K. A. (2011). Detecting and mapping vegetation distribution on the Antarctic Peninsula from remote sensing data. Polar Biol. 34, 273–281. 10.1007/s00300-010-0880-2

[B16] Fretwell P. T. LaRue M. A. Morin P. Kooyman G. L. Wienecke B. Ratcliffe N.. (2012). An emperor penguin population estimate: the first global, synoptic survey of a species from space. PLoS ONE 7:e33751. 10.1371/annotation/32c246eb-3b73-4410-a44c-b41ddae11fc5 22514609 PMC3325796

[B17] Fujii M. Takano Y. Kojima H. Hoshino T. Tanaka R. Fukui M. (2010). Microbial community structure, pigment composition, and nitrogen source of red snow in antarctica. Microb. Ecol. 59, 466–475. 10.1007/s00248-009-9594-9 19847476 PMC4261141

[B18] Ganey G. Q. Loso M. G. Burgess A. B. Dial R. J. (2017). The role of microbes in snowmelt and radiative forcing on an Alaskan icefield. Nat. Geosci. 10, 754–759. 10.1038/ngeo3027

[B19] Gray A. Krolikowski M. Fretwell P. Convey P. Peck L. S. Mendelova M.. (2020). Remote sensing reveals Antarctic green snow algae as important terrestrial carbon sink. Nat. Commun. 11:2527. 10.1038/s41467-020-16018-w 32433543 PMC7239900

[B20] Hirano M. (1965). Freshwater algae in the Antarctic regions, in Biogeography and Ecology in Antarctica, Chapter 4, eds van Mieghem J. van Oye P. (Heidelberg: Springer Science & Business Media), 127–193. 10.1007/978-94-015-7204-0_4

[B21] Hodson A. J. Anesio A. M. Tranter M. Fountain A. G. Osborn M. Priscu J. C.. (2008). Glacial ecosystems. Ecol. Monogr. 78, 41–67. 10.1890/07-0187.1

[B22] Hodson A. J. Nowak A. Cook J. Sabacka M. Wharfe E. S. Pearce D. A.. (2017). Microbes influence the biogeochemical and optical properties of maritime Antarctic snow. J. Geophys. Res. Biogeosci. 122, 1456–1470. 10.1002/2016JG003694

[B23] Hoham R. W. Duval B. (2001). Microbial ecology of snow and freshwater ice with emphasis on snow algae, in Snow Ecology: An Interdisciplinary Examination of Snow-Covered Ecosystems, eds Jones H. G. Pomeroy J. W. Walker D. A. Hoham R. W. (Cambridge: Cambridge University Press), 168–228.

[B24] Hoham R. W. Remias D. (2020). Snow and glacial algae: a review. J. Phycol. 56, 264–282. 10.1111/jpy.12952 31825096 PMC7232433

[B25] Holzinger A. Allen M. C. Deheyn D. D. (2016). Hyperspectral imaging of snow algae and green algae from aeroterrestrial habitats. J. Photochem. Photobiol. B Biol. 162, 412–420. 10.1016/j.jphotobiol.2016.07.001 27442511 PMC5061078

[B26] Howat I. M. Porter C. Smith B. E. Noh M.-J. Morin P. (2019). The reference elevation model of Antarctica. Cryosphere 13, 665–674. 10.5194/tc-13-665-2019

[B27] Huovinen P. Ramírez J. Gómez I. (2018). Remote sensing of albedo-reducing snow algae and impurities in the Maritime Antarctica. ISPRS J. Photogramm. Rem. Sens. 146, 507–517. 10.1016/j.isprsjprs.2018.10.015

[B28] Jawak S. D. Luis A. J. Fretwell P. T. Convey P. Durairajan U. A. (2019). Semiautomated detection and mapping of vegetation distribution in the antarctic environment using spatial-spectral characteristics of WorldView-2 imagery. Rem. Sens. 11:1909. 10.3390/rs11161909

[B29] Khan A. L. Dierssen H. Scambos T. Höfer J. Cordero R. R. (2020). Spectral characterization, radiative forcing, and pigment content of coastal antarctic snow algae: approaches to spectrally discriminate red and green communities and their impact on snowmelt. Cryosphere Discuss. 2020, 1–27. 10.5194/tc-2020-170

[B30] Khan A. L. Dierssen H. M. Scambos T. A. Höfer J. Cordero R. R. (2021). Spectral characterization, radiative forcing and pigment content of coastal antarctic snow algae: approaches to spectrally discriminate red and green communities and their impact on snowmelt. Cryosphere 15, 133–148. 10.5194/tc-15-133-2021

[B31] Kokhanovsky A. Di Mauro B. Garzonio R. Colombo R. (2021). Retrieval of dust properties from spectral snow reflectance measurements. Front. Environ. Sci. 9:644551. 10.3389/fenvs.2021.644551

[B32] LaRue M. A. Rotella J. J. Garrott R. A. Siniff D. B. Ainley D. G. Stauffer G. E.. (2011). Satellite imagery can be used to detect variation in abundance of Weddell seals (*Leptonychotes weddellii*) in Erebus Bay, Antarctica. Polar Biol. 34, 1727–1737. 10.1007/s00300-011-1023-0

[B33] Ling H. U. Seppelt R. D. (1993). Snow algae of the Windmill Islands continental Antarctica. 2. *Chloromonas rubroleosa* sp. nov. (Volvocales, Chlorophyta), an alga of red snow. Eur. J. Phycol. 28, 77–84. 10.1080/09670269300650131

[B34] Lutz S. Anesio A. M. Raiswell R. Edwards A. Newton R. J. Gill F.. (2016). The biogeography of red snow microbiomes and their role in melting arctic glaciers. Nat. Commun. 7:11968. 10.1038/ncomms11968 27329445 PMC4917964

[B35] Malard L. A. Šabacká M. Magiopoulos I. Mowlem M. Hodson A. Tranter M.. (2019). Spatial variability of Antarctic surface snow bacterial communities. Front. Microbiol. 10:461. 10.3389/fmicb.2019.00461 30972032 PMC6443967

[B36] Marshall W. Chalmers M. (1997). Airborne dispersal of Antarctic terrestrial algae and cyanobacteria. Ecography 20, 585–594. 10.1111/j.1600-0587.1997.tb00427.x

[B37] Mataloni G. Tesolín G. (1997). A preliminary survey of cryobiontic algal communities from Cierva Point (Antarctic Peninsula). Antarctic Sci. 9, 250–258. 10.1017/S0954102097000333

[B38] Muller T. Thomas L. Fuhr G. (2001). Persistent snow algal fields in Spitsbergen: field observations and a hypothesis about the annual cell circulation. Arctic Antarctic Alpine Res. 33, 42–51. 10.1080/15230430.2001.12003403

[B39] Nolin A. W. (2011). Recent advances in remote sensing of seasonal snow. J. Glaciol. 56, 1141–1150. 10.3189/002214311796406077

[B40] Nowak A. Hodson A. Turchyn A. V. (2018). Spatial and temporal dynamics of dissolved organic carbon, chlorophyll, nutrients, and trace metals in Maritime Antarctic snow and snowmelt. Front. Earth Sci. 6:201. 10.3389/feart.2018.00201

[B41] Painter T. H. Duval B. Thomas W. H. Mendez M. Heintzelman S. Dozier J. (2001). Detection and quantification of snow algae with an airborne imaging spectrometer. Appl. Environ. Microbiol. 67, 5267–5272. 10.1128/AEM.67.11.5267-5272.2001 11679355 PMC93300

[B42] Perovich D. K. (2007). Light reflection and transmission by a temperate snow cover. J. Glaciol. 53, 201–210. 10.3189/172756507782202919

[B43] Procházková L. Leya T. Krížková H. Nedbalová L. (2019). *Sanguina nivaloides* and *Sanguina aurantia* gen. et spp. Nov. (Chlorophyta): The taxonomy, phylogeny, biogeography and ecology of two newly recognised algae causing red and orange snow. FEMS Microbiol. Ecol. 95:fiz064. 10.1093/femsec/fiz064 31074825 PMC6545352

[B44] Radoux J. Chomé G. Jacques D. C. Waldner F. Bellemans N. Matton N.. (2016). Sentinel-2's potential for sub-pixel landscape feature detection. Rem. Sens. 8:488. 10.3390/rs8060488

[B45] Remias D. Wastian H. Lütz C. Leya T. (2013). Insights into the biology and phylogeny of *Chloromonas polyptera* (Chlorophyta), an alga causing orange snow in Maritime Antarctica. Antarctic Sci. 25, 648–656. 10.1017/S0954102013000060

[B46] Segawa T. Matsuzaki R. Takeuchi N. Akiyoshi A. Navarro F. Sugiyama S.. (2018). Bipolar dispersal of red-snow algae. Nat. Commun. 9:3094. 10.1038/s41467-018-05521-w 30082897 PMC6079020

[B47] Soto D. F. Fuentes R. Huovinen P. Gómez I. (2020). Microbial composition and photosynthesis in Antarctic snow algae communities: integrating metabarcoding and pulse amplitude modulation fluorometry. Algal Res. 45:101738. 10.1016/j.algal.2019.101738

[B48] Takeuchi N. Dial R. Kohshima S. Segawa T. Uetake J. (2006). Spatial distribution and abundance of red snow algae on the Harding Icefield, Alaska derived from a satellite image. Geophys. Res. Lett. 33, 1–6. 10.1029/2006GL027819

[B49] Tedstone A. Cook J. Williamson C. Hofer S. McCutcheon J. Irvine-Fynn T.. (2019). Algal growth and weathering crust structure drive variability in Greenland Ice Sheet ice albedo. Cryosphere Discuss. 14, 521–538. 10.5194/tc-14-521-2020

[B50] Vermote E. F. Tanre D. Deuze J. L. Herman M. Morcette J. (1997). Second simulation of the satellite signal in the solar spectrum 6s: an overview. IEEE Trans. Geosci. Rem. Sens. 35, 675–686. 10.1109/36.581987

[B51] Weyermann J. Schläpfer D. Hueni A. Kneubühler M. Schaepman M. (2009). Spectral Angle Mapper (SAM) for anisotropy class indexing in imaging spectrometry data, in Imaging Spectrometry XIV, Vol. 7457, eds Shen S. S. Lewis P. E. (San Diego, CA: SPIE), 74570B. 10.1117/12.825991

